# Induction of Brain Tumors by the Archetype Strain of Human Neurotropic JCPyV in a Transgenic Mouse Model

**DOI:** 10.3390/v13020162

**Published:** 2021-01-22

**Authors:** Luis Del Valle, Kamel Khalili

**Affiliations:** 1 Neurological Cancer Research, Stanley S. Scott Cancer Center, Departments of Medicine and Pathology, Louisiana State University Health, New Orleans, LA 70112, USA; 2 Department of Neurosciences and Center for Neurovirology, Lewis Katz School of Medicine at Temple University, Philadelphia, PA 19140, USA

**Keywords:** CNS tumors, JCPyV, T-Antigen, transgenic mice, primitive neuroectodermal tumor, pituitary tumor, malignant peripheral nerve sheath tumor, glioblastoma

## Abstract

JC Virus (JCPyV), a member of the *Polyomaviridiæ* family, is a human neurotropic virus with world-wide distribution. JCPyV is the established opportunistic infectious agent of progressive multifocal leukoencephalopathy, a fatal demyelinating disease, which results from the cytolytic infection of oligodendrocytes. Mutations in the regulatory region of JCPyV determine the different viral strains. Mad-1 the strain associated with PML contains two 98 base pair repeats, whereas the archetype strain (CY), which is the transmissible form of JCPyV, contains only one 98 tandem with two insertions of 62 and 23 base pairs respectively. The oncogenicity of JCPyV has been suspected since direct inoculation into the brain of rodents and primates resulted in the development of brain tumors and has been attributed to the viral protein, T-Antigen. To further understand the oncogenicity of JCPyV, a transgenic mouse colony containing the early region of the archetype strain (CY), under the regulation of its own promoter was generated. These transgenic animals developed tumors of neural crest origin, including: primitive neuroectodermal tumors, medulloblastomas, adrenal neuroblastomas, pituitary tumors, malignant peripheral nerve sheath tumors, and glioblastomas. Neoplastic cells from all different phenotypes express T-Antigen. The close parallels between the tumors developed by these transgenic animals and human CNS tumors make this animal model an excellent tool for the study of viral oncogenesis.

## 1. Introduction

JC Virus (JCPyV), a human neurotropic virus, member of the *Polyomaviridiae* family, which also includes BKPyV, SV40, and the more recently discovered Merkel Cell Polyomavirus (MCPyV) [1], is the established etiological agent of the fatal demyelinating disease progressive multifocal leukoencephalopathy (PML). JCPyV circulates widely among the human population of the world, as demonstrated by many epidemiological and serological studies [2,3]. Two, not mutually exclusive routes of transmission have been postulated. A respiratory infection is based on the finding of JCPyV genomic sequences in tonsillar stromal cells and B-lymphocytes [4,5], and a fecal-oral route of infection has gained notoriety based on the presence of live viral particles in raw urban sewage in different countries [6,7], and/or viral genomic sequences in the epithelial cells of the gastrointestinal tract [8,9]. In addition and supporting this theory, JCPyV has been found contaminating raw oysters [10]. Infection with JCPyV produces no symptomatology and is thought to occur in early childhood [3]. After a subclinical primary infection, the virus is thought to remain in a latent state preferentially in the kidney [11,12], until its reactivation under immunosuppressive conditions, to result in PML [13,14]. Before the AIDS pandemic PML was a rare disease, usually associated with leukemias, lymphomas, or transplant immunosuppression. However after the first report of PML in an HIV-infected individual [15], the high prevalence of this demyelinating disease in AIDS patients has made PML an AIDS-defining condition [16]. The demyelinating plaques characteristic of the disease are the result of the active infection and lytic destruction of oligodendrocytes by actively replicating JCPyV. Other hallmarks of PML include eosinophilic intranuclear inclusion bodies in infected oligodendrocytes and the presence of atypical, bizarre astrocytes, which morphologically resemble neoplastic cells of astrocytic origin [17,18].

### 1.1. JCPyV Genomic Structure

**Figure 1:**
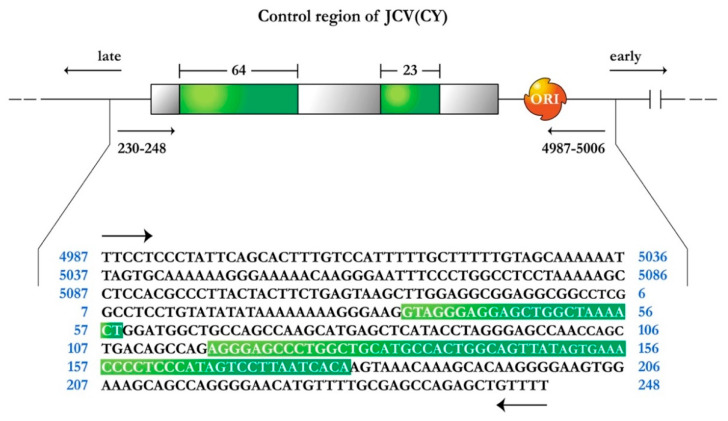
Schematic representation of the JC Virus archetype strain CY, control region. Green boxes indicate the 64 and 23 base pair sequences that are not present in the Mad1 strain within the 98 base pair promoter/enhancer sequence, now divided into three empty gray boxes of 25, 55, and 18 base pairs. Arrows indicate the direction of early and late gene transcription. ORI represents the origin for viral DNA replication. The nucleotide sequence of the control region is shown below, depicting the 64 and 23 insertions (green boxes).

The genome of JCPyV consists of a circular, closed, double stranded DNA, and is composed of 5130 nucleotides. It is comprised of three regions; an early coding region, which contains the genes for non-structural regulatory proteins small t-antigen and large T-Antigen, a late transcriptional region, which encodes for the capsid proteins VP1, VP2 and VP3, and the small accessory product Agnoprotein. These two coding regions are separated by a non-coding regulatory region, which contains the early and late promoters as well as the site of viral DNA replication [19,20]. Variations within the regulatory region are observed in the different strains of JCPyV. The prototype strain of JCPyV or Mad-1, which is isolated from the brain of individuals with PML and is responsible for the active infection of oligodendrocytes, has a promoter/enhancer region comprised of two 98 base pair repeats [19]. Another variant also isolated from cases of PML, Mad-4, also contains two 98 base pair repeats, however, the second tandem is truncated to 79 base pairs. On the other hand, the archetype strain or CY, which remains in latent state in the kidney, has been isolated from the urine of healthy individuals and is thought to be the transmissible form of JCPyV, containing only one 98 base pair tandem, with two insertions of 64 and 23 base pairs respectively, and divides the 98 base pair region into three segments of 25, 55, and 18 base pairs respectively, as depicted in Figure 1 [21]. It would appear that deletions of the 23 and 64 nucleotide sequences from the CY strain and duplication of the remaining 98 bp create the control region present in the Mad strains. Furthermore, these and other rearrangements in the control region may also be responsible for cellular tissue tropism, host range, and for the activation of JCPyV from a latent to an active infection.

### 1.2. JCPyV Oncogenicity

Since the original description of PML by Åström and Richardson [22,23], the discovery of viral particles in oligodendrocytes from PML cases [24,25] and the isolation of virions in culture [26], several experimental animal models were developed by directly injecting viral particles into the brain, in an effort to reproduce the demyelinating lesions of PML. However, none of the inoculated animals developed plaques of myelin loss, and surprisingly the majority of them developed brain tumors of different phenotypes. Intracerebral inoculation of the Mad-1 strain of JCPyV in golden hamsters at birth resulted in the development of glioblastomas, ependymomas, and primitive neuroectodermal tumors (PNETs), including medulloblastomas, 3 to 6 months post-inoculation [27,28]. Interestingly, intra-ocular inoculation of JCPyV into neonate hamsters, resulted in the development of adrenal neuroblastomas [29], suggesting the high tropism of JCPyV for neuroectodermal-derived structures. Inoculation of JCPyV Mad-1 into the brain of newborn rats resulted in the development of cortical PNETs at the site of viral introduction [30,31]. A different strain of JCPyV, Tokyo-1, also resulted in the development of PNETs and medulloblastomas when inoculated into the brain of hamsters [32]. Finally, intracerebral injection of the Mad-1 strain of JCPyV, into owl and squirrel monkeys resulted in the development of glial origin neoplasms, ranging from astrocytomas in a few cases to Glioblastomas in the majority of the animals [33,34].

Since then, the transforming abilities of T-Antigen have been demonstrated in vitro [35], and later, the association of JCPyV with human brain tumors has been extensively studied and documented. JCPyV DNA sequences and viral protein expression in neoplastic cells have been shown in a wide variety of tumors originating in the central nervous system, including tumors of neural origin such as medulloblastomas [36,37,38], glial tumors ranging from low grade astrocytomas, to anaplastic gliomas and glioblastoma multiforme [39,40,41,42], and in ependymal tumors [40,43]. In most cases, either the Mad-1 or the Mad-4 strains have been identified in association with human brain neoplasms. Interestingly, in one study, the archetype strain has been amplified and sequenced from human brain tumors, in two cases of oligodendroglioma [44].

In order to further understand the oncogenicity of JCPyV, a colony of transgenic mice was created, containing the early region of the archetype strain of JCPyV (CY) under the control of its own promoter/regulatory region. Since these transgenic animals contain exclusively the early coding region, the oncogenicity can only be attributed to the expression of the early product, T-Antigen. In the present manuscript we histologically and immunohistochemically characterize and summarize the different types of tumors developed by these transgenic animals.

## 2. Materials and Methods

### 2.1. Development of Transgenic Mice

Transgenic mice were generated using the methodology originally described by Gordon and Ruddle [45,46]. Briefly, a 3.2 kilobase Bal1/NciI restriction fragment, which contained the early coding region of the archetype strain of JCPyV (CY), including only the promoter/enhancer region were injected into fertilized mouse oocytes generated by FVB/N mouse mating. In order to identify the mice carrying the transgene, genomic DNA was isolated from the tail and analyzed by polymerase chain reaction, utilizing JCPyV control region specific primers. Amplification was performed with one set of primers, from 4987–5006 (5′-TTCCTCCCTATTCAGCACTT-3′), and 230–248 (5′-AAAACAGCTCTGGCTCGCAA-3′), which flanks the 400 bp control region. Figure 1 depicts a schematic representation of the JCPyV archetype (CY) control region.

### 2.2. Histology and Immunohistochemistry

Tissues were fixed in 10% buffered formalin and embedded in paraffin. Sections of 4–5 microns in thickness were cut and placed on electromagnetically charged slides (Fisher Scientific, Fair Lawn, NJ, USA) to prevent detachment. Hematoxylin and Eosin was performed for routine histopathological examination. Tumors were diagnosed according to the latest World Health Organization Classification of Tumors of the Nervous System [47]. Immunohistochemistry was performed using the avidin-biotin-peroxidase methodology, according to the manufacturer’s instructions (Vectastain ABC Elite Kit, Vector Laboratories, Burlingame, CA, USA). Our modified protocol includes deparaffination in xylenes, rehydration through descending grades of ethanol up to water, non-enzymatic antigen retrieval with 0.01 M sodium citrate buffer pH 6.0 at 95 °C for 25 min, endogenous peroxidase quenching with 3% H_2_O_2_ in methanol, blocking with normal horse serum (for mouse monoclonal antibodies) or normal goat serum (for rabbit polyclonal or recombinant rabbit monoclonal antibodies) and incubation with primary antibodies overnight at room temperature in a humidified chamber. Antibodies for viral proteins included a mouse monoclonal anti-SV40 T-Antigen, which cross reacts with the T-Antigen of JCPyV (clone PAb416, 1:100 dilution, Millipore/Sigma, Burlington, MA, USA); p53 was detected with a mouse monoclonal antibody (Clone DO-7, DAKO/Agilent, Santa Clara, CA, USA); antibodies for cellular markers included mouse monoclonals against Class III-beta Tubulin (Clone TuJ1, 1:500 dilution, Novus Biologicals Centennial, CO, USA), Chromogranin A (Clone DAK-A3, 1:200 dilution, DAKO/Agilent), S-100 (Clone B32.1, 1:500 dilution, abcam, Cambridge, MA, USA), Neuron-Specific Enolase (Clone BBS/NC/VI-H14, 1:500 dilution, DAKO/Agilent), Glial Fibrillary Acidic Protein (GFAP) (Clone SMI25, 1:1000 dilution, Sternberger/Biolegend, San Diego, CA, USA), and a recombinant rabbit monoclonal antibody against Prolactin (EPR18018-31, 1:16,000 dilution, abcam). After rinsing in PBS, sections were incubated with biotinylated secondary antibodies for 1 h, followed by incubation with avidin-biotin-peroxidase complexes for 1 h, both at room temperature in a humidified chamber. Finally, the peroxidase was developed with diaminobenzidine (Boehringer, Mannheim, Germany) for 3 min, and the sections were counterstained with Hematoxylin and mounted with Permount (Fisher Scientific). Photomicrographs were taken with an Olympus DP72 Digital Camera using an Olympus BX70 microscope (Olympus, Center Valley, PA, USA).

### 2.3. Double Labeling Immunofluorescence

The first part of our protocol for double labeling is similar to the methodology described above, with the exception of endogenous peroxidase quenching step, which was not performed. However, after incubation with the first primary antibody (T-Antigen), an Alexa Flour 488-conjugated anti-mouse secondary antibody was incubated for 1 h in the dark. Sections were then washed thoroughly with PBS, blocked again, and a second primary antibody raised in a different species than the first one (recombinant rabbit monoclonal anti-p53, 1:100 dilution, DAKO/Agilent), was incubated overnight. Finally, a second Alexa Fluor 568-conjugated anti-rabbit secondary antibody was incubated for 1 h in the dark and slides were cover-slipped with an aqueous mounting media without DAPI (Vectashield Plus Antifade, Vector Laboratories), since both proteins were expected in the nucleus, and visualized in an Olympus FV100 confocal microscope.

### 2.4. Protein Extraction and Western Blot Analysis

Direct Western blot analysis was performed on crude protein extracts from tumors and various other organs, including the brain, heart, lung, liver, spleen, and kidney from all phenotypes. Protein extracts were obtained from 500 μg of tissue homogenized in TNN buffer (50 mmol/L Tris pH 7.4, 50 mmol/L NaCl, and 0.5% Nonidet P-40) with a mammalian protease inhibitor cocktail at 1% (SIGMA-Aldrich, St. Louis, MO, USA). Homogenized tissues were centrifuged at 14,000 rpm at 4 °C for 5 min and protein concentration was determined using the NanoDrop ND-1000 spectrophotometer (ND-1000 V.3.8 software, https://nanodrop-1000.software.informer.com/3.8/) for each supernatant. Equal amounts of protein extracts (50 μg) were separated using a 10% acrylamide gel and transferred to a pure nitrocellulose membrane (Trans-blot Transfer Medium, Bio-Rad Laboratories Inc, Hercules, CA, USA). The membrane was then blocked in 10% milk in PBS-T (0.01% Tween 20). A monoclonal mouse anti-SV40 T-Antigen antibody (clone pAb416) was incubated overnight at 4 °C at a 1:250 dilution, and an anti-mouse horseradish peroxidase-conjugated secondary antibody (Pierce, Rockford, IL, USA) was used at a 1:5000 dilution. Primary and secondary antibodies were diluted in 5% milk in PBS-T. Visualization of proteins was performed using the Amersham ECL Plus Detection System (GE Healthcare, Buckinghamshire, UK) according to the manufacturer’s instructions. Later, the membrane was stripped using stripping buffer (2% SDS, 5 mmol/L Na_2_HPO_4_, pH 7.5) at 60 °C for 45 min, washed and incubated with a mouse monoclonal anti-GRB2 antibody (clone 81, 1:500 dilution, BD Biosciences, San Jose, CA, USA).

## 3. Results

The final number of transgenic mice after 10 years was 1560. Of these, a total of 240 developed tumors for a 15.38%. The distribution of these tumors by phenotype is shown in Table 1 below. These mice started showing neurological signs and symptoms between 9 and 13 months of age, which included ataxia, hunched posture, inability to properly groom and in the late stages paralysis of the rear limbs, and histopathological examination showed the presence of poorly differentiated, small-blue cell tumors in the cerebellum, consistent with medulloblastomas and supratentorial tumors consistent with primitive neuroectodermal tumors [48]. Later it was shown that these tumors contained populations of cells that exhibit robust expression of T-Antigen, while others have weak levels and others are completely negative. Interestingly, in positive cells T-Antigen was found in association with wild-type p53 and pRb, but in negative cells a novel p53 mutation involving a deletion between residues 35 and 153 was detected and characterized [49]. After that, other tumor phenotypes started to appear, all of them derived from the neuroectoderm, including pituitary tumors and malignant peripheral nerve sheath tumors [50,51].

### Characterization of Tumors in CY Archetype Transgenic Mice

**Figure 2:**
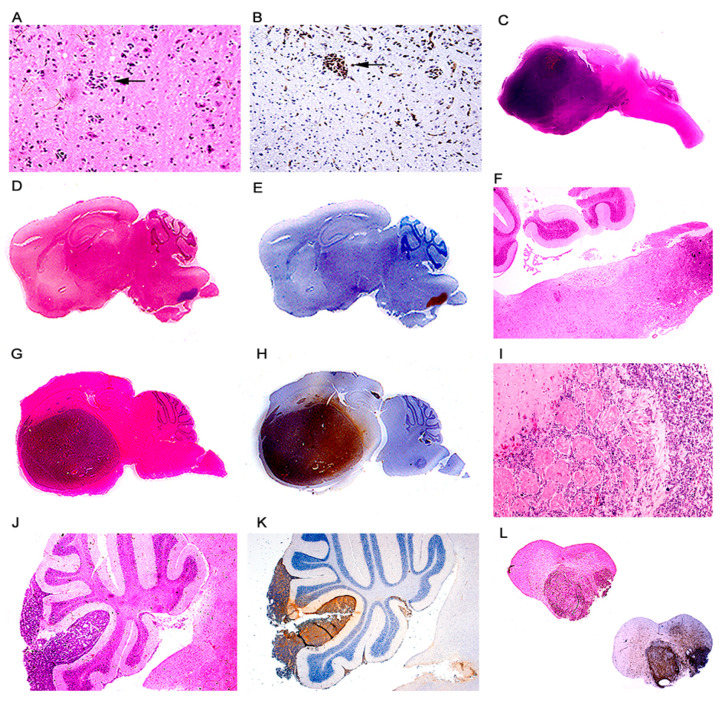
Primitive neuroectodermal tumors; location. Low magnification view of a JCPyV transgenic mouse brain demonstrating a small pocket of neoplastic cells (arrow) in the lower portion of the brainstem ((**A**) hematoxylin and eosin). T-Antigen immunolabeling highlights this small collection of neoplastic cells (100× magnification). (**B**). Full montage of a JCPyV transgenic mouse brain, demonstrating a large tumor, which substitutes the majority of the brain parenchyma (100× magnification). ((**C**) H & E). Consecutive full montages showing very small PNETs located in the brainstem ((**D**,**E**) H & E and T-Antigen respectively). At low magnification the small blue cell tumors in the medulla are evident (Panel **F**). These embryonal tumors can also reach large dimensions when located in the basal ganglia ((**G**,**H**) H & E and T-Antigen respectively). In very few cases the tumors originated in the olfactory bulb ((**I**) 100× magnification). Panels (**J**) and (**K**) depict the subarachnoid dissemination of these tumors in the cerebellum, typical of PNETs. Finally, very few cases de-veloped tumors in the spinal cord ((**L**) full montage, H & E and T-Antigen).

*Primitive Neuroectodermal Tumors (PNETs).* This phenotype was by far the most frequently observed in the archetype strain transgenic animals. 178 developed PNETs, all of them between 2 and 6 months after birth. The tumors ranged in size from microscopic foci of few neoplastic cells (Figure 2A,B), to giant masses that substitute close to 80% of the brain parenchyma (Figure 2C). The tumors originated in different locations of the central nervous system. The most frequent location observed has been the lower part of the brainstem and upper portion of the cervical spinal cord with 69 animals developing tumors in this place (Figure 2D–H), followed by the basal ganglia with 52 (Figure 2J,K), the cerebral hemispheres, especially the frontal and parietal cortices with 34. Tumors arising from the cerebellum, which were classified as medulloblastomas accounted for 8. Tumors in the spinal cord appeared in 7 animals, and more rarely the tumors arise from the hippocampus (4), and the olfactory bulb also with 4 cases (Figure 2I). Macroscopically, the tumors appear as poorly circumscribed, infiltrating masses, of solid consistency and gray color, with no discernable areas of necrosis or hemorrhage, and very similar in appearance to medulloblastomas in humans. Table 2 below shows the location of these embryonal tumors.

**Figure 3:**
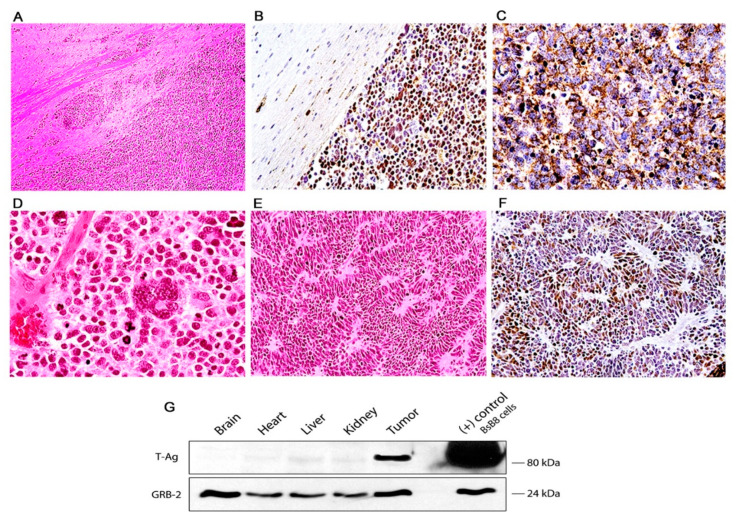
Primitive neuroectodermal tumors in JCV transgenic mice; histological and immunohistochemical characterization. The tumors are composed of sheaths of numerous small and densely packed cells, which diffusely infiltrate the brain parenchyma (Panel (**A**), hematoxylin and eosin, original magnification ×100). Immunohistochemistry for JCPyV T-antigen highlights the presence of the primitive neoplastic cells (Panel (**B**) original magnification ×100). Immunohistochemistry for Class III β-tubulin reveals the early neural origin of the tumors (Panel (**C**), original magnification ×200). At higher magnification the slightly elongated nuclei and scanted cytoplasm of the cells can be appreciated. Numerous mitotic figures and apoptotic bodies are present as well as giant multinucleated cells (Panel (**D**), hematoxylin and eosin, original magnification ×400). The neoplastic cells frequently arrange in a circular, concentric pattern, which resembles Homer-Wright rosette formation. Neoplastic cells in these rosette formations express T-Antigen (Panels (**E**) and (**F**), H&E and T-Antigen respectively, original magnification ×100). (**G**) Western blot from protein extracts of different organs of the transgenic mice, probed with T-Antigen demonstrates the presence of the oncoprotein in the tumor (Medulloblastoma), but its absence in all organs, including the normal brain adjacent to the tumor.

Histologically, the tumors are characterized by sheaths of numerous small and densely packed, basophilic cells. These neoplastic cells have slightly elongated nuclei, and possess scant cytoplasm, giving them a poorly differentiated appearance. Mitotic figures and apoptotic bodies are very frequently observed (Figure 3D). Giant multinucleated cells, with the same nuclear characteristics are present with regularity in the tumors, and are presumably the result of the high mitotic activity rate of the neoplastic cells. Homer-Wright rosettes were found with regularity with certain predilection for tumors originated in the basal ganglia (Figure 3E,F). Subarachnoid dissemination of neoplastic cells was observed in 19 cases (Figure 2L) which is the preferential route of spreading for medulloblastomas in humans.

**Figure 4:**
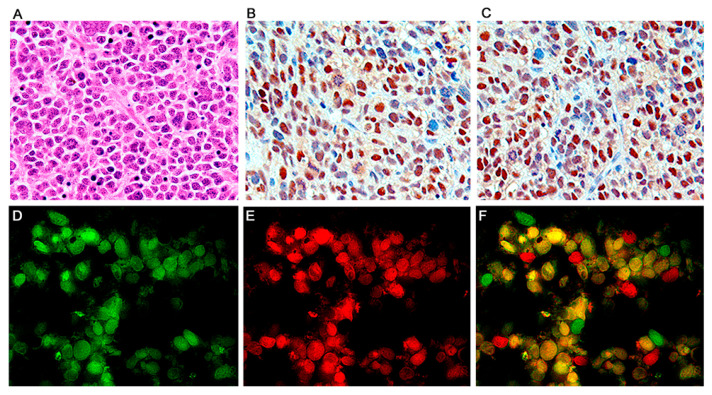
Primitive neuroectodermal tumors; T-Antigen and cell cycle proteins. High magnification view of a PNET, formed by sheaths of numerous small basophilic cells, where mitotic figures and apoptosis bodies can be seen (Panel (**A**), hematoxylin and eosin, original magnification ×600). Immunohistochemistry for T-Antigen demonstrates the nuclear localization of the protein in approximately 75–80% of the neoplastic cells. (Panel (**B**)). p53 expression was robustly found in the nuclei of approximately the same number of neoplastic cells in all PNETs (Panel (**C**)). Immunofluorescence with a fluorescein tagged antibody for T-Antigen reveals robust nuclear signal in neoplastic cells (Panel (**D**)); p53, tagged with rhodamine is also expressed in the nuclei of the primitive neoplastic cells (Panel (**E**)). Double labeling, utilizing a deconvolution microscope reveals the co-localization of both proteins in the nuclear compartment (Panel (**F**), all panels original magnification ×1000).

By immunohistochemistry, early neural cellular markers were consistently expressed. Class III β-tubulin was found in the cytoplasm of the neoplastic cells in all the cases (Figure 3C), while expression of synaptophysin was found in approximately 65% of the tumors. Very importantly, JCPyV early protein, T-Antigen is robustly expressed in all the tumors, but interestingly, it is present in approximately 65–70% of neoplastic cells, while a small subpopulation of these tumor cells remains negative (Figure 4B). Although these are transgenic mice, in which every single cell of every tissue contains the transgene, expression of T-Antigen is limited to the tumors, and not even the adjacent brain tissue shows the presence of the oncogenic protein, as demonstrated in a representative Western blot from a PNET (Figure 3G).

**Figure 5:**
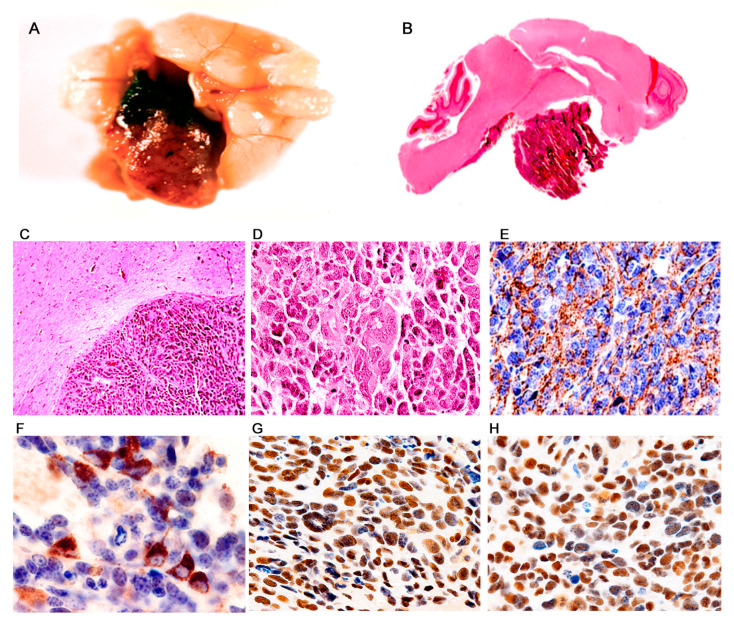
Pituitary tumors; histological and immunohistochemical characterization. Macroscopically, pituitary tumors were large, well circumscribed, solid, brown, masses located in the diencephalic area (Panel (**A**)). Montage showing the extra-axial homogeneous tumor with a prominent vascular network (Panel (**B**)). Low power view of a well-circumscribed, extra-axial neoplasm, which does not infiltrate the brain parenchyma (Panel (**C**), H&E, original magnification ×100). At higher magnification the tumor is composed of numerous pleomorphic cells with irregular nuclei and abundant eosinophilic cytoplasm. Giant multinucleated cells are present (Panel (**D**), H&E, original magnification ×400). Immunohistochemistry for chromogranin shows extensive cytoplasmic expression in neoplastic cells (Panel (**E**), original magnification ×200). Occasionally, neoplastic cells showed robust immunoreactivity for prolactin (Panel (**F**), original magnification ×400). Expression of the JCPyV early product, T-Antigen was found in the nuclei of numerous neoplastic cells, including giant multinucleated cells (Panel (**G**)), and p53 was also detected in the nuclei of tumor cells (Panel (**H**), both panels original magnification ×400).

*Pituitary Tumors.* This phenotype was observed in 21 of the transgenic animals. The tumors were located at the base of the brain, sitting and expanding the *Sella turca*, and they presented as well circumscribed and solid masses of dark brown to dark red and purple color and firm consistency. The tumors were extra-axial and pushed, but did not infiltrate the suprajacent brain parenchyma (Figure 5A). Histologically the tumors are characterized by numerous homogeneous cells with a round, relatively large nucleus and abundant eosinophilic cytoplasm. The neoplastic cells ranged in size from medium to large and occasionally multinucleated giant cells were observed (Figure 5B). Immunohistochemically the majority of neoplastic cells in all the tumors demonstrated cytoplasmic expression of chromogranin (Figure 5C), and in some cells from all fourteen cases, prolactin (Figure 5D) confirming the pituitary-cell origin of these tumors. Similar to the PNETs, all the pituitary tumors express T-Antigen in the majority, but not all of neoplastic cells, strongly suggesting a “hit-and-run” mechanism. As it was in the case with PNETs and medulloblastomas, only the tumor expressed T-Antigen and neither other organs or the adjacent brain showed any expression of T-Antigen which speaks of the high neurotropism of JCPyV.

**Figure 6:**
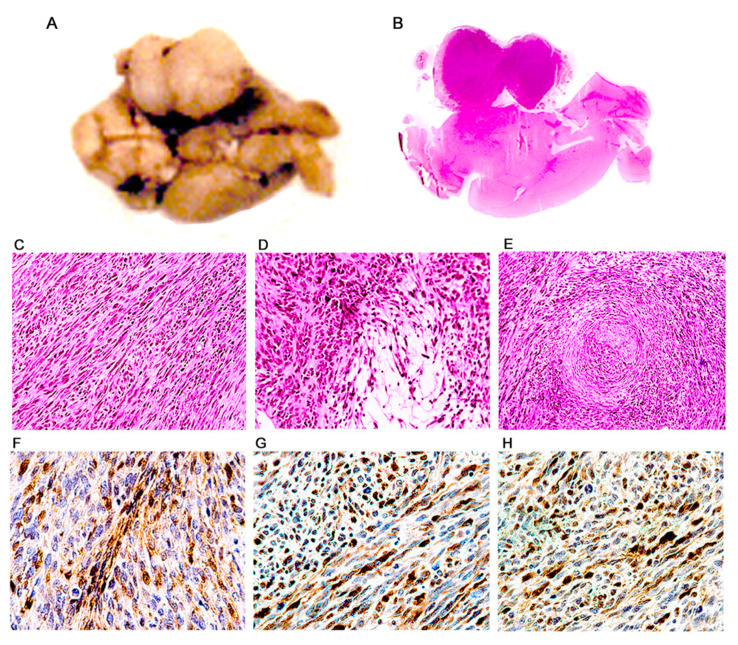
Malignant peripheral nerve sheath tumors (MPNSTs); histological and immunohistochemical characterization. Ventral surface of a transgenic mouse brain showing a large, well-circumscribed, solid, homogeneous mass located in the ponto-cerebellar angle (Panel (**A**)). A full montage demonstrates that the tumor is extra-axial and located in the place of the vestibulocochlear nerve (Panel (**B**)). Histologically, tumors are composed of numerous, tightly packed, spindle cells, which form interlacing bundles and fascicles. A large number of mitotic figures are present (Panel (**C**)). Dense cellularity areas (Antoni type A) alternate with areas where cells are loosely distributed (Antoni type B) (Panel (**D**)). Also present are cellular whirling areas, which correspond to Verocay bodies (Panel (**E**)). Immunohistochemistry for S-100 protein shows intense reactivity in the cytoplasm of neoplastic cells (Panel (**F**)). T-Antigen (Panel (**G**)), and p53 (Panel (**H**)) are expressed in the nuclei of neoplastic cell throughout the tumors. (Panels (**B**)–(**E**), hematoxylin & eosin all panels original magnification ×400).

*Malignant Peripheral Nerve Sheath Tumors.* A total of 18 mice have developed this phenotype. The tumors were located mainly in the extremities, in the subcutaneous region of the dorsal and lateral abdominal wall and in the wall of the rib cage, presumably originating from intercostal nerves, and another frequent location was intracranial in the site of the VIII cranial nerve (Figure 6A,B). Macroscopically, the tumors were well circumscribed, homogeneous, ranging from white to yellowish-gray in color, of very firm consistency, and with a fasciculate appearance. Histologically, these tumors were composed of numerous spindle cells, with an elongated and wavy nucleus and abundant cytoplasm. Mitotic activity was abundant (Figure 6C). The neoplastic cells are tightly packed and form bundles and interdigitating fascicles. With regularity, we found areas of myxoid degeneration where neoplastic cells are loosely distributed (Antoni type B areas), adjacent to areas of densely packed cells (Antoni type A areas) (Figure 6D). Also, but with less frequency we found areas where the neoplastic cells form concentric swirls, corresponding to Verocay bodies (Figure 6E). Immunohistochemistry demonstrated extensive expression of S-100 protein, confirming the peripheral nerve sheath origin of the tumors (Figure 6F).

**Figure 7:**
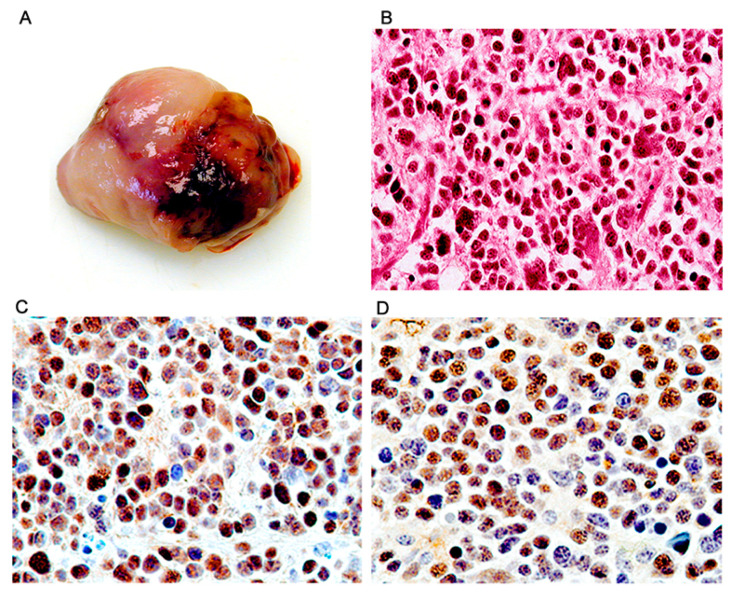
Adrenal neuroblastomas; histological and immunohistochemical characterization. Tumors originated in the adrenal gland and were grossly solid, homogeneous mases (Panel **A**). Histologically the tumors were composed of sheets of round cells exhibiting nuclear pleomorphism and scant cytoplasm (Panel **B**, hematoxylin and eosin). By immunohistochemistry, the majority of neoplastic cells showed robust expression of T-Antigen (Panel **C**), and wild-type p53 (Panel **D**). (All panels original magnification ×400).

*Adrenal neuroblastomas.* Similarly to PNETs, adrenal neuroblastomas developed early, between 2 and 5 months after birth. All of the 7 tumors were located in the abdominal cavity and while the majority were very large masses that filled about half of the abdominal cavity there was evidence they originated in the upper portion of one kidney, including a smaller tumor, which clearly originated in the adrenal gland. In all instances the tumors were infiltrating adjacent organs, including the wall of the intestine. The tumors were solid, well circumscribed, and encapsulated, and their inner part was homogenous, yellow greyish, firm to the touch and well vascularized (Figure 7A). Histologically, the tumors were composed of sheets of poorly differentiated round cells with large round nuclei and scant to moderated cytoplasm and exhibited some degree of nuclear atypia and occasionally multinucleation (Figure 7B). By immunohistochemistry, the tumors expressed early neuronal markers, including neuron specific enolase (NSE), synaptophysin, and Class III β-tubulin. T-Antigen was robustly expressed in the nuclei of the majority of tumor cells in all tumors, and like in all other phenotypes, expression of wild-type p53 was also present in the same compartment (Figure 7C,D, respectively).

**Figure 8:**
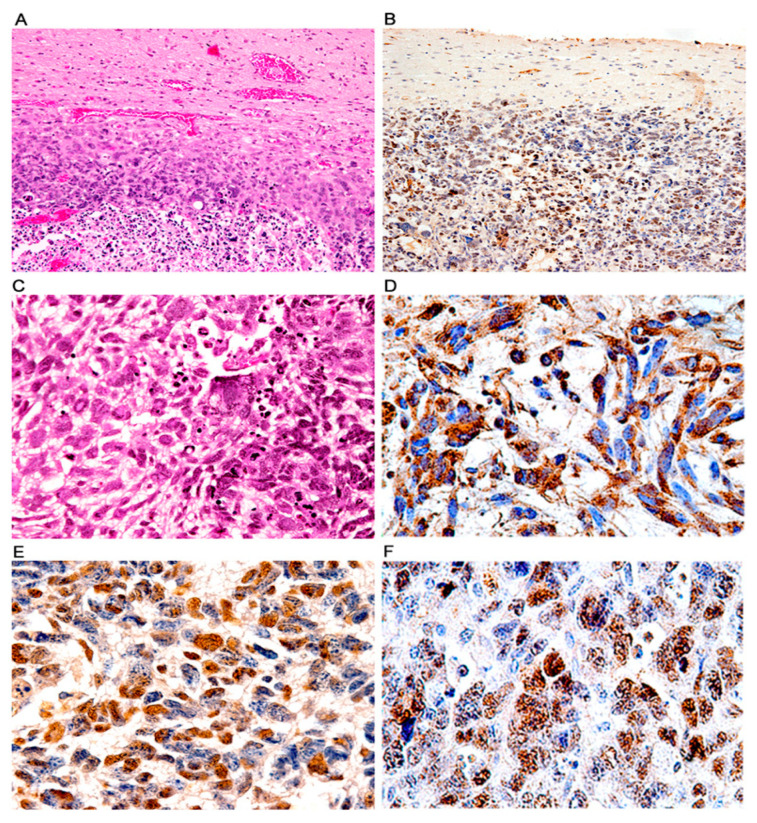
Glioblastomas; histological and immunohistochemical characterization. Low magnification views of glial tumors originating in the cortex of transgenic mice. These tumors exhibit prominent areas of necrosis (Panel (**A**), H&E), and T-Antigen expression (Panel (**B**), both panels original magnification 100×). Histologically, these aggressive tumors were composed of numerous irregular malignant cells with highly atypical and pleomorphic nuclei and abundant cytoplasm. Numerous giant multinucleated cells, mitotic figures and areas of necrosis are observed (Panel (**C**), hematoxylin and eosin). Robust cytoplasmic immunoreactivity for GFAP is present in practically all neoplastic cells confirming the astrocytic phenotype (Panel (**D**)). Expression of T-Antigen (Panel (**E**)) and p53 is found in the nuclei of neoplastic astrocytes (Panel (**F**)). (Panels (**C**)–(**F**) original magnification ×400).

*Glioblastomas.* While this phenotype is the less frequent by far with only 4 mice developing glioblastomas, its significance is high because the tumors closely resemble their human counterparts. The tumors presented as very large, poorly circumscribed cortical hemispheric masses that diffusely infiltrated the brain parenchyma (Figure 8A,B, H&E and T-Antigen, respectively). Histologically the tumors were composed of numerous pleomorphic cells with atypical nuclei and abundant eosinophilic cytoplasm over a dense fibrillary background. Giant multinucleated cells and mitotic activity were prominent (Figure 8C). Areas of necrosis were found throughout the tumors, and neoplastic cells surrounding these necrotic areas gave the typical “pseudo-palisading” appearance. Immunohistochemistry against glial fibrillary acidic protein (GFAP) confirmed the astrocytic phenotype of the tumors as the majority of neoplastic cells expressed the glial specific intermediate filament (Figure 8D). Finally, expression of T-Antigen was present in the nuclei of approximately 70% of neoplastic cells (Figure 8E), and the cell cycle regulator p53, wild-type was also present in these cells (Figure 8F).

*Multiple Phenotypes.* Interestingly, 12 animals have developed tumors with two different phenotypes at the same time. All the combinations involved PNETs. In 5 of them we found the concomitant presence of PNETs, all of them located to the brainstem, with malignant peripheral nerve sheath tumors of the extremities. 7 others developed PNETs in the brainstem, olfactory bulb or basal ganglia, and pituitary tumors.

*Non-Neural Tumors.* Finally, 6 mice developed other unusual, non-neural-related neoplasms. Two of them developed angiosarcomas, one originated in a lower extremity and the second originated in the liver. Five mice developed lymphomas, present in the spleen, liver, and peripheral nodes. Two mice developed angiosarcomas, one in the rear extremity and one in the abdominal cavity. One mouse developed a papillary carcinoma of the thyroid gland and one mouse presented with an endodermic sack tumor of the ovary. Expression of T-Antigen was consistently absent in these tumors by immunohistochemistry (not shown), and for this reason we presume these are sporadic tumors that are not related to the presence of the JCPyV transgene.

## 4. Discussion

The first clues on the oncogenic potential of the human neurotropic virus JCPyV originated in early experimental models aimed to reproduce the demyelinating disease PML in rodents and monkeys. Surprisingly, injection of JCPyV particles resulted, not in demyelination, but in the development of a variety of neuroectodermal origin tumors, including PNETs, medulloblastomas, and glial neoplasms (ependymomas and astrocytomas). Although the development of brain tumors after intracerebral inoculation constitutes direct and irrefutable evidence of the oncogenicity of JCPyV, the mechanism of carcinogenesis remained unknown for some time. However, in the last four decades, mounting evidence has been generated pointing to the early protein T-Antigen as the protein responsible for de-regulating several pathways important for controlling cell proliferation. JCPyV T-Antigen is a multifunctional protein composed of 688 amino-acids, transcribed during the early phase of infection, before viral DNA replication, important for the regulation of the JCPyV life cycle. Among its functions, T-Antigen plays a central role in the initiation of JCPyV replication, unwinding of DNA at the site of origin, and eventually represses early transcription and promotes late gene transcription [52]. However, T-Antigen has also some unfortunate effects for cells harboring JCPyV genome in the absence of viral replication. It contains pRb- and p53-binding domains, which are capable to sequester and inactivate these two key cell cycle regulator proteins. Binding and stabilization of p53 will slow cell cycle progression and prevent apoptosis, a scenario that although beneficial for viral replication, will also facilitate malignant transformation [53,54]. T-Antigen has also shown to physically bind to β-catenin [55], and to IRS-1 [56,57], translocating these normally cytoplasmic proteins to the nuclear compartment and also contributing to malignant transformation. It is known that de-regulation of both pathways plays a role in the development of medulloblastomas among other neoplasms. In addition, T-Antigen has shown to cause chromosomal aberrations in circulating lymphocytes [58] in glioblastoma cell lines [59], either by itself, or in combination with other proteins, and to disrupt the cellular DNA repair machinery [60,61].

For decades JCPyV was the only human polyomavirus proven to be oncogenic in animal models and suspected in humans. The development of these tumors of neuroectodermal origin in our transgenic animal model containing the early, large T-Antigen transcriptional region JCPyV opened the door for the search of this ubiquitous and widespread DNA virus in human brain tumors, which resulted in the detection of integrated viral genomic sequences in human medulloblastomas [36,37,38], and eventually in human glial tumors [40]. More recently, the discovery of the novel Merkel cell Polyomavirus (MCPyV) and its clear role in the pathogenesis of Merkel cell carcinomas [62] opened new insights into the oncogenicity of polyomaviruses in humans. Now the role of JCPyV and specifically of the oncoprotein T-Antigen have been demonstrated also in colon carcinomas [9,55,63], and another notorious human polyomavirus, BKPyV has been recently implicated in the oncogenesis of urothelial and bladder carcinomas [64,65,66]. This animal model has also yielded valuable data to elucidate the different oncogenic pathways affected by T-Antigen. For example, the physical binding and inactivation of p53 was corroborated in the pituitary tumors, event that results in the accumulation of the p53 downstream target responsible for cell cycle progression, P21/WAF [50]. In PNETs it was shown that E2F, the transcription factor that dissociates from pRb and stimulates S-phase-specific genes is highly expressed when T-Antigen is bound to pRb [67]. In the case of the malignant peripheral nerve sheath tumors, it was shown that T-Antigen was binding and inactivating NF2, the putative tumor suppressor protein associated with neurofibromatosis type 2. Interestingly, T-Antigen was capable of translocating NF2, a normally cytoplasmic protein into the nucleus. The consequences of this translocation beyond the inactivation of NF2 are yet to be studied [51].

One constant observation in all the tumors and thought all phenotypes is the variability in nuclear T-Antigen expression. While some cells exhibit robust expression, other cells demonstrate much lower levels and other cells are completely negative, and fascinatingly, these cells can be located next to each other. These observations have contributed to the “hit-and-run” theory [68]. In this theory, it is proposed that initial infection leads to a series of transforming events, such as chromosomal breakages and damage [58], activation of anti-apoptotic pathways, which will prevent the destruction of virally infected cells [69,70], dysregulation of cell cycle regulatory pathways, such as p53, pRb, the Wnt signaling pathway [71], and other; however, once the malignant transformation has occurred, the maintenance of T-Antigen requires significant amounts of energy, and JCPyV-infected/transformed cells shut down the transcription of T-Antigen [72] and JCPyV then faces natural selection pressures that cease the expression of T-Antigen. Corroborating this theory, from the PNETs developed by these transgenic mice, positive and negative cells were isolated and cloned and several experiments conducted. When inoculated into the flank of nude mice, T-Antigen positive cells grow much larger tumors and at a much faster rate than their T-Antigen negative counterparts. Another interesting observation is that a large number of mice did not develop any tumors and T-Antigen was not expressed. The reason why the transgene was not activated in these mice is not known; we can only hypothesize that the multiple transcription factors that drive the early promoter were not active, or perhaps the immune system response to cells expressing T-Antigen or early transformed cells could have prevented tumor formation. Further studies would be necessary to answer these questions.

In recent years a breakthrough discovery has refreshed the views on the brain oncogenic properties of polyomaviruses. While all animal models related to JCPyV have been experimental, a new polyomavirus was discovered in naturally occurring glioblastomas in racoons, animals that usually do not develop glial tumors [73]. Furthermore, while clustered, none of the raccoons were not genetically related, and all the tumors occurred in the frontal lobe along the sulci of the olfactory nerve, suggesting that the novel racoon polyomavirus was environmental and the medium of transmission was respiratory. Expression of T-Antigen was detected in tumor cells in a similar pattern to that in humans, with intensely positive cells mixed with lower expressing and negative cells [74]. These findings in the only naturally occurring brain tumor caused by a neurotropic polyomavirus in animals, validate all the JCPyV findings in our experimental transgenic mice and in human oncogenesis by JCPyV.

## 5. Conclusions

Animal models have traditionally played a key role in studying human diseases, however, because of its narrow tissue and species tropism, conferred by its control region, there has been a lack of animal models to study the effects of JCPyV infection, and specifically of T-Antigen, making this transgenic model ideal for studying the effects of T-Antigen in its natural state, since the transgene contains its own promoter. Interestingly, the neoplasms developed by these transgenic mice are all derived from the neuroectoderm, corroborating the high neurotropism of JCPyV, and included primitive neuroectodermal tumors, cerebellar medulloblastomas, pituitary tumors, adrenal neuroblastomas, malignant peripheral nerve sheath tumors, and glioblastomas. The most frequent phenotype observed by far is the PNET, which also appeared between 2 and 6 months compared to the 9 to 12 months for all the other better differentiated tumors, suggesting that the time of T-Antigen expression during development and its early impact in deregulating different signaling pathways is crucial in the development of particular phenotypes, and mirroring humans where medulloblastomas are usually tumors of childhood. The specific expression of T-Antigen in the absence of capsid formation or viral replication and the high number of tumors developed in a natural setting make this transgenic model ideal for the studying JCPyV oncogenicity.

## Figures and Tables

**Table 1:** Tumor phenotypes developed by JCPyV transgenic mice. Characterization of the tumors was based on the World Health Organization Classification of Brain Tumors. Other phenotypes included 5 lymphomas, 2 angiosarcomas, 1 papillary carcinoma of the thyroid, and 1 endodermal sinus tumor.

Primitive Neuroectodermal Tumors and Medulloblastomas	Pituitary Tumors	Malignant Peripheral Nerve Sheath Tumors	Adrenal Neuroblastomas	Glioblastomas
178	21	18	7	4
	**PNET + Pituitary**	**PNET + MPNST**		
	5	7		

**Table 2:** Location of primitive neuroectodermal tumors developed in JCPyV (CY) transgenic mice. In 23 cases the tumors involved both the cerebral cortex and the basal ganglia. In 5 cases the tumors involved all parts of the forebrain. 19 cases demonstrated subarachnoid dissemination. Tumors with this phenotype originating in the cerebellum were classified as medulloblastomas.

Primitive Neuroectodermal Tumors (178)						
Spinal Cord	Brainstem	Cerebellum Medulloblastomas	Basal Ganglia	Cerebral Hemispheres	Hippocampus	Olfactory Bulb
7	69	8	52	34	4	4

## Data Availability

Data is contained within the article.

## References

[1] Moens U., Calvignac-Spencer S., Lauber C., Ramqvist T., Feltkamp M.C.W., Daugherty M.D., Verschoor E.J., Ehlers B., Ictv Report C. (2017). ICTV Virus taxonomy profile: Polyomaviridae. J. Gen. Virol..

[2] Walker D.L., Padgett B.L. (1983). The epidemiology of human Polyomaviruses. Prog. Clin. Biol Res..

[3] Taguchi F., Kajioka J., Miyamura T. (1982). Prevalence rate and age of acquisition of antibodies against JC Virus and BK Virus in human sera. Microbiol. Immunol..

[4] Monaco M.C., Jensen P.N., Hou J., Durham L.C., Major E.O. (1998). Detection of JC virus DNA in human tonsil tissue: Evidence for site of initial viral infection. J. Virol..

[5] Wei G., Liu C.K., Atwood W.J. (2000). JC virus binds to primary human glial cells, tonsillar stromal cells, and B-lymphocytes, but not to T lymphocytes. J. NeuroVirol..

[6] Bofill-Mas S., Pina S., Girones R. (2000). Documenting the epidemiologic patterns of Polyomaviruses in human populations by studying their presence in urban sewage. Appl. Env. Microbiol..

[7] Bofill-Mas S., Formiga-Cruz M., Clemente-Casares P., Calafell F., Girones R. (2001). Potential transmission of human Polyomaviruses through the gastrointestinal tract after exposure to virions or viral DNA. J. Virol..

[8] Ricciardiello L., Laghi L., Ramamirtham P., Chang C.L., Chang D.K., Randolph A.E., Boland C.R. (2000). JC Virus DNA sequences are frequently present in the human upper and lower gastrointestinal tract. Gastroenterology.

[9] Laghi L., Randolph A.E., Chauhan D.P., Marra G., Major E.O., Neel J.V., Boland C.R. (1999). JC Virus DNA is present in the mucosa of the human colon and in colorectal cancers. Proc. Natl. Acad. Sci. USA.

[10] Abreu I.N., Cortinhas J.M., Dos Santos M.B., Queiroz M.A.F., da Silva A., Cayres-Vallinoto I.M.V., Vallinoto A.C.R. (2020). Detection of Human Polyomavirus 2 (HPyV2) in oyster samples in northern Brazil. Virol. J..

[11] Coleman D.V., Daniel R.A., Gardner S.D., Field A.M., Gibson P.E. (1977). Polyoma Virus in urine during pregnancy. Lancet.

[12] Markowitz R.B., Eaton B.A., Kubik M.F., Latorra D., McGregor J.A., Dynan W.S. (1991). BK Virus and JC Virus shed during pregnancy have predominantly archetypal regulatory regions. J. Virol..

[13] Major E.O., Amemiya K., Tornatore C.S., Houff S.A., Berger J.R. (1992). Pathogenesis and molecular biology of Progressive Multifocal Leukoencephalopathy, the JC virus-induced demyelinating disease of the human brain. Clin. Microbiol. Rev..

[14] Berger J.R., Concha M. (1995). Progressive Multifocal Leukoencephalopathy: The evolution of a disease once considered rare. J. NeuroVirol..

[15] Miller J.R., Barrett R.E., Britton C.B., Tapper M.L., Bahr G.S., Bruno P.J., Marquardt M.D., Hays A.P., McMurtry J.G., Weissman J.B. (1982). Progressive Multifocal Leukoencephalopathy in a male homosexual with T-cell immune deficiency. N. Engl. J. Med..

[16] Holman R.C., Torok T.J., Belay E.D., Janssen R.S., Schonberger L.B. (1998). Progressive Multifocal Leukoencephalopathy in the United States, 1979–1994: Increased mortality associated with HIV infection. Neuroepidemiology.

[17] Aksamit A.J. (1995). Progressive Multifocal Leukoencephalopathy: A review of the pathology and pathogenesis. Microsc. Res. Tech..

[18] Del Valle L., Piña-Oviedo S. (2006). HIV disorders of the brain: Pathology and pathogenesis. Front. Biosci..

[19] Frisque R.J., Bream G.L., Cannella M.T. (1984). Human Polyomavirus JC virus genome. J. Virol..

[20] Raj G.V., Khalili K. (1995). Transcriptional regulation: Lessons from the human neurotropic Polyomavirus, JCV. Virology.

[21] Yogo Y., Kitamura T., Sugimoto C., Ueki T., Aso Y., Hara K., Taguchi F. (1990). Isolation of a possible archetypal JC virus DNA sequence from non-immunocompromised individuals. J. Virol..

[22] Åström K.E., Mancall E.L., Richardson E.P. (1958). Progressive Multifocal Leuko-encephalopathy; a hitherto unrecognized complication of chronic lymphatic leukaemia and Hodgkin’s disease. Brain.

[23] Richardson E.P. (1961). Progressive Multifocal Leukoencephalopathy. N. Engl. J. Med..

[24] Silverman L., Rubinstein L.J. (1965). Electron microscopic observations on a case of Progressive Multifocal Leukoencephalopathy. Acta NeuroPathol..

[25] Zu Rhein G., Chou S.M. (1965). Particles resembling Papova viruses in human cerebral demyelinating disease. Science.

[26] Padgett B.L., Walker D.L., Zu Rhein G.M., Eckroade R.J., Dessel B.H. (1971). Cultivation of Papova-like virus from human brain with Progressive Multifocal Leucoencephalopathy. Lancet.

[27] Walker D.L., Padgett B.L., Zu Rhein G.M., Albert A.E., Marsh R.F. (1973). Human Papovavirus (JC): Induction of brain tumors in hamsters. Science.

[28] Zu Rhein G.M., Varakis J.N. (1979). Perinatal induction of medulloblastomas in Syrian golden hamsters by a human polyoma virus (JC). Natl. Cancer Inst. Monogr..

[29] Varakis J., Zu Rhein G.M., Padgett B.L., Walker D.L. (1978). Induction of peripheral neuroblastomas in Syrian hamsters after injection as neonates with JC virus, a human polyoma virus. Cancer Res..

[30] Ohsumi S., Ikehara I., Motoi M., Ogawa K., Nagashima K., Yasui K. (1985). Induction of undifferentiated brain tumors in rats by a human Polyomavirus (JC virus). Jpn. J. Cancer Res..

[31] Ohsumi S., Motoi M., Ogawa K. (1986). Induction of undifferentiated tumors by JC Virus in the cerebrum of rats. Acta Pathol. Jpn..

[32] Nagashima K., Yasui K., Kimura J., Washizu M., Yamaguchi K., Mori W. (1984). Induction of brain tumors by a newly isolated JC virus (Tokyo-1 strain). Am. J. Pathol..

[33] London W.T., Houff S.A., Madden D.L., Fuccillo D.A., Gravell M., Wallen W.C., Palmer A.E., Sever J.L., Padgett B.L., Walker D.L. (1978). Brain tumors in owl monkeys inoculated with a human Polyomavirus (JC virus). Science.

[34] London W.T., Houff S.A., McKeever P.E., Wallen W.C., Sever J.L., Padgett B.L., Walker D.L. (1983). Viral-induced astrocytomas in squirrel monkeys. Prog. Clin. Biol Res..

[35] O’Neill F.J., Frisque R.J., Xu X., Hu Y.X., Carney H. (1995). Immortalization of human cells by mutant and chimeric primate polyomavirus T-Antigen genes. Oncogene.

[36] Khalili K., Krynska B., Del Valle L., Katsetos C.D., Croul S. (1999). Medulloblastomas and the human neurotropic polyomavirus JC virus. Lancet.

[37] Krynska B., Del Valle L., Croul S., Gordon J., Katsetos C.D., Carbone M., Giordano A., Khalili K. (1999). Detection of human neurotropic JC virus DNA sequence and expression of the viral oncogenic protein in pediatric medulloblastomas. Proc. Natl. Acad Sci. USA.

[38] Del Valle L., Gordon J., Enam S., Delbue S., Croul S., Abraham S., Radhakrishnan S., Assimakopoulou M., Katsetos C.D., Khalili K. (2002). Expression of human neurotropic Polyomavirus JCV late gene product Agnoprotein in human Medulloblastoma. J. Natl. Cancer Inst..

[39] Caldarelli-Stefano R., Boldorini R., Monga G., Meraviglia E., Zorini E.O., Ferrante P. (2000). JC Virus in human glial-derived tumors. Hum. Pathol..

[40] Del Valle L., Gordon J., Assimakopoulou M., Enam S., Geddes J.F., Varakis J.N., Katsetos C.D., Croul S., Khalili K. (2001). Detection of JC Virus DNA sequences and expression of the viral regulatory protein T-Antigen in tumors of the Central Nervous System. Cancer Res..

[41] Del Valle L., Azizi S.A., Krynska B., Enam S., Croul S.E., Khalili K. (2000). Reactivation of human neurotropic JC Virus expressing oncogenic protein in a recurrent Glioblastoma multiforme. Ann. Neurol..

[42] Piña-Oviedo S., De Leon-Bojorge B., Cuesta-Mejias T., White M.K., Ortiz-Hidalgo C., Khalili K., Del Valle L. (2006). Glioblastoma multiforme with small cell neuronal-like component: Association with human neurotropic JC Virus. Acta NeuroPathol..

[43] Okamoto H., Mineta T., Ueda S., Nakahara Y., Shiraishi T., Tamiya T., Tabuchi K. (2005). Detection of JC Virus DNA sequences in brain tumors in pediatric patients. J. Neurosurg..

[44] Del Valle L., Enam S., Lara C., Ortiz-Hidalgo C., Katsetos C.D., Khalili K. (2002). Detection of JC Polyomavirus DNA sequences and cellular localization of T-Antigen and Agnoprotein in Oligodendrogliomas. Clin. Cancer Res..

[45] Gordon J.W., Scangos G.A., Plotkin D.J., Barbosa J.A., Ruddle F.H. (1980). Genetic transformation of mouse embryos by microinjection of purified DNA. Proc. Natl. Acad. Sci. USA.

[46] Gordon J.W., Ruddle F.H. (1981). Integration and stable germ line transmission of genes injected into mouse pronuclei. Science.

[47] Louis D.N., Perry A., Reifenberger G., von Deimling A., Figarella-Branger D., Cavenee W.K., Ohgaki H., Wiestler O.D., Kleihues P., Ellison D.W. (2016). The 2016 World Health Organization Classification of Tumors of the Central Nervous System: A summary. Acta NeuroPathol..

[48] Krynska B., Otte J., Franks R., Khalili K., Croul S. (1999). Human ubiquitous JCV(CY) T-Antigen gene induces brain tumors in experimental animals. Oncogene.

[49] Krynska B., Del Valle L., Gordon J., Otte J., Croul S., Khalili K. (2000). Identification of a novel p53 mutation in JCV-induced mouse medulloblastoma. Virology.

[50] Gordon J., Del Valle L., Otte J., Khalili K. (2000). Pituitary neoplasia induced by expression of human neurotropic Polyomavirus, JCV, early genome in transgenic mice. Oncogene.

[51] Shollar D., Del Valle L., Khalili K., Otte J., Gordon J. (2004). JCV T-Antigen interacts with the neurofibromatosis type 2 gene product in a transgenic mouse model of malignant peripheral nerve sheath tumors. Oncogene.

[52] White M.K., Khalili K. (2004). Polyomaviruses and human cancer: Molecular mechanisms underlying patterns of tumorigenesis. Virology.

[53] Dyson N., Bernards R., Friend S.H., Gooding L.R., Hassell J.A., Major E.O., Pipas J.M., Vandyke T., Harlow E. (1990). Large T-Antigens of many Polyomaviruses are able to form complexes with the Retinoblastoma protein. J. Virol..

[54] Levine A.J. (1997). p53, the cellular gatekeeper for growth and division. Cell.

[55] Enam S., Del Valle L., Lara C., Gan D.D., Ortiz-Hidalgo C., Palazzo J.P., Khalili K. (2002). Association of human Polyomavirus JCV with colon cancer: Evidence for interaction of viral T-Antigen and beta-catenin. Cancer Res..

[56] Del Valle L., Enam S., Lassak A., Wang J.Y., Croul S., Khalili K., Reiss K. (2002). Insulin-like growth factor I receptor activity in human medulloblastomas. Clin. Cancer Res..

[57] Lassak A., Del Valle L., Peruzzi F., Wang J.Y., Enam S., Croul S., Khalili K., Reiss K. (2002). Insulin receptor substrate 1 translocation to the nucleus by the human JC Virus T-Antigen. J. Biol. Chem..

[58] Neel J.V., Major E.O., Awa A.A., Glover T., Burgess A., Traub R., Curfman B., Satoh C. (1996). Hypothesis: “Rogue cell”-type chromosomal damage in lymphocytes is associated with infection with the JC human polyoma virus and has implications for oncopenesis. Proc. Natl. Acad. Sci. USA.

[59] Tognon M., Casalone R., Martini F., De Mattei M., Granata P., Minelli E., Arcuri C., Collini P., Bocchini V. (1996). Large T-Antigen coding sequences of two DNA tumor viruses, BK and SV40, and nonrandom chromosome changes in two Glioblastoma cell lines. Cancer Genet. Cytogenet..

[60] Trojanek J., Croul S., Ho T., Wang J.Y., Darbinyan A., Nowicki M., Del Valle L., Skorski T., Khalili K., Reiss K. (2006). T-Antigen of the human Polyomavirus JC attenuates faithful DNA repair by forcing nuclear interaction between IRS-1 and Rad51. J. Cell Physiol..

[61] Darbinyan A., White M.K., Akan S., Radhakrishnan S., Del Valle L., Amini S., Khalili K. (2007). Alterations of DNA damage repair pathways resulting from JCV infection. Virology.

[62] Feng H., Shuda M., Chang Y., Moore P.S. (2008). Clonal integration of a Polyomavirus in human Merkel cell carcinoma. Science.

[63] Ricciardiello L., Chang D.K., Laghi L., Goel A., Chang C.L., Boland C.R. (2001). Mad-1 is the exclusive JC Virus strain present in the human colon, and its transcriptional control region has a deleted 98-base-pair sequence in colon cancer tissues. J. Virol..

[64] Tognon M., Corallini A., Martini F., Negrini M., Barbanti-Brodano G. (2003). Oncogenic transformation by BK Virus and association with human tumors. Oncogene.

[65] Muller D.C., Ramo M., Naegele K., Ribi S., Wetterauer C., Perrina V., Quagliata L., Vlajnic T., Ruiz C., Balitzki B. (2018). Donor-derived, metastatic urothelial cancer after kidney transplantation associated with a potentially oncogenic BK polyomavirus. J. Pathol..

[66] Zeng Y., Sun J., Bao J., Zhu T. (2020). BK polyomavirus infection promotes growth and aggressiveness in bladder cancer. Virol. J..

[67] Krynska B., Gordon J., Otte J., Franks R., Knobler R., DeLuca A., Giordano A., Khalili K. (1997). Role of cell cycle regulators in tumor formation in transgenic mice expressing the human neurotropic virus, JCV, early protein. J. Cell Biochem..

[68] Ferreira D.A., Tayyar Y., Idris A., McMillan N.A.J. (2020). A “hit-and-run” affair—A possible link for cancer progression in virally driven cancers. Biochim. Biophys. Acta Rev. Cancer.

[69] Piña-Oviedo S., Urbanska K., Radhakrishnan S., Sweet T., Reiss K., Khalili K., Del Valle L. (2007). Effects of JC Virus infection on anti-apoptotic protein Survivin in Progressive Multifocal Leukoencephalopathy. Am. J. Pathol..

[70] Del Valle L., Sweet T., Parker-Struckhoff A., Perez-Liz G., Pina-Oviedo S. (2020). JCPyV T-Antigen activation of the anti-apoptotic Survivin promoter—Its role in the development of Progressive Multifocal Leukoencephalopathy. Viruses.

[71] Ripple M.J., Parker Struckhoff A., Trillo-Tinoco J., Li L., Margolin D.A., McGoey R., Del Valle L. (2014). Activation of c-Myc and cyclin D1 by JCV T-Antigen and beta-catenin in colon cancer. PLoS ONE.

[72] Donadoni M., Sariyer R., Wollebo H., Bellizzi A., Sariyer I.K. (2018). Viral tumor antigen expression is no longer required in radiation-resistant subpopulation of JCV induced mouse medulloblastoma cells. Genes Cancer.

[73] Dela Cruz F.N., Giannitti F., Li L., Woods L.W., Del Valle L., Delwart E., Pesavento P.A. (2013). Novel Polyomavirus associated with Brain Tumors in Free-Ranging Raccoons, Western United States. Emerg. Infect. Dis..

[74] Brostoff T., Dela Cruz F.N., Church M.E., Woolard K.D., Pesavento P.A. (2014). The Raccoon Polyomavirus genome and tumor antigen transcription are stable and abundant in neuroglial tumors. J. Virol..

